# Extensive deep vein thrombosis in a young girl with absent inferior vena cava and iliac veins: a case report

**DOI:** 10.1093/omcr/omad008

**Published:** 2023-02-27

**Authors:** Gufran Algaly, Ali Alsuyihili, Ayesha Parveen

**Affiliations:** Emergency Medicine, Hamad Medical Corporation, Doha, Qatar; Internal Medicine, Hamad Medical Corporation, Doha, Qatar; Emergency Medicine, Hamad Medical Corporation, Doha, Qatar

## Abstract

Congenital absence of inferior vena cava (IVC) and iliac veins is a rare anomaly that can predispose young patients to develop deep vein thrombosis (DVT). This case report highlights the importance of considering this anatomical abnormality in young patients with unprovoked DVT. We present the case of a 17-year-old girl who visited the emergency department (ED) complaining of right leg pain and swelling for 8 days. An ED ultrasound revealed extensive DVT in the right leg veins, and further investigation with abdominal computed tomography revealed that the patient’s IVC and iliac veins were absent and showed the presence of thrombosis. The patient underwent thrombectomy and angioplasty by interventional radiology and was given a lifetime prescription for oral anticoagulation. When treating young, otherwise healthy individuals with unprovoked DVT, Clinicians should include absent IVC in their differential when treating young, otherwise healthy patients with unprovoked DVT.

## INTRODUCTION

Congenital absence of inferior vena cava (IVC) is an extremely rare condition. However, it poses an important risk factor for developing deep vein thrombosis (DVT) in young patients, despite the presence of other venous collaterals [1]. Furthermore, 5% of young patients under 30 years described having absent IVC, indicating that congenital absence of IVC may not be an infrequent finding in young patients with DVT [2].

## CASE REPORT

A previously healthy 17-year-old female presented to our emergency department (ED) with an 8-day history of right lower limb swelling and pain, which progressed gradually. The pain was so severe that she was not able to bear weight. She denied fever, oral contraceptive use, recent travel or trauma.

On clinical examination, there was significant swelling over the right limb from the foot extending to the thigh. The limb felt very tense, with an intact distal neurovascular examination. An X-ray taken showed no bony abnormalities. Doppler ultrasound showed evidence of thrombus filling the common femoral vein, superficial femoral vein, popliteal vein and proximal posterior tibial vein with the extension of the thrombus into the right external vein, and partial compression, sluggish flow, and subcutaneous edema.

The patient was started on anticoagulant treatment in ED and then admitted under medicine for a full workup. All labs, including autoimmune screening, flow cytometry and hemolysis screening, were negative (Table 1).

**Table 1:** Blood investigation results

Group	Detail	Value w/units
Hepatitis serology	Hepatitis B surface antigen	Non-reactive
Hepatitis serology	Hepatitis C Ab	Non-reactive
HIV and HTLV serology	HIV Ag/Ab combo	Non-reactive
General immunology	c3	1.98 gm/l
General immunology	C4	0.62 gm/l
Autoimmune diseases	ANCA	Negative
Autoimmune diseases	Anti-cardiolipin Ab IgG	1.30 GPL
Autoimmune diseases	Anti-ardiolipin Ab IgG Int	Negative
Autoimmune diseases	Anti-cardiolipin Ab IgM	0.80>MPL
Autoimmune diseases	Anti-cardiolipin Ab IgM Int	Negative
Autoimmune diseases	Anti-B2 glycoprotein IgG	1.30 U/ml
Autoimmune diseases	Anti-B2 glycoprotein IgG Int	Negative
Autoimmune diseases	Anti-B2 glycoprotein IgM	2.90 >U/ml
Autoimmune diseases	Anti-B2 glycoprotein IgM Int	Negative
Autoimmune diseases	ANA CTD Int	Negative

**Figure 1:**
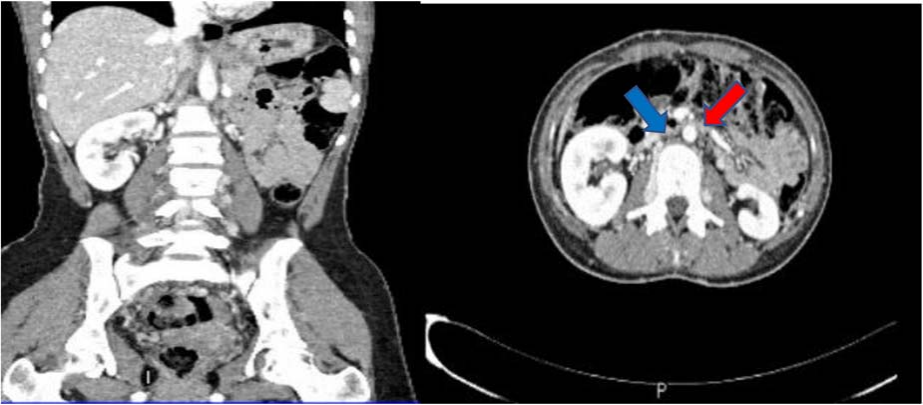
Sagittal and coronal views, absence of IVC at suprarenal and infrarenal levels, the red arrow showing the aorta and the blue arrow indicating where the IVC should typically be located.

**Figure 2:**
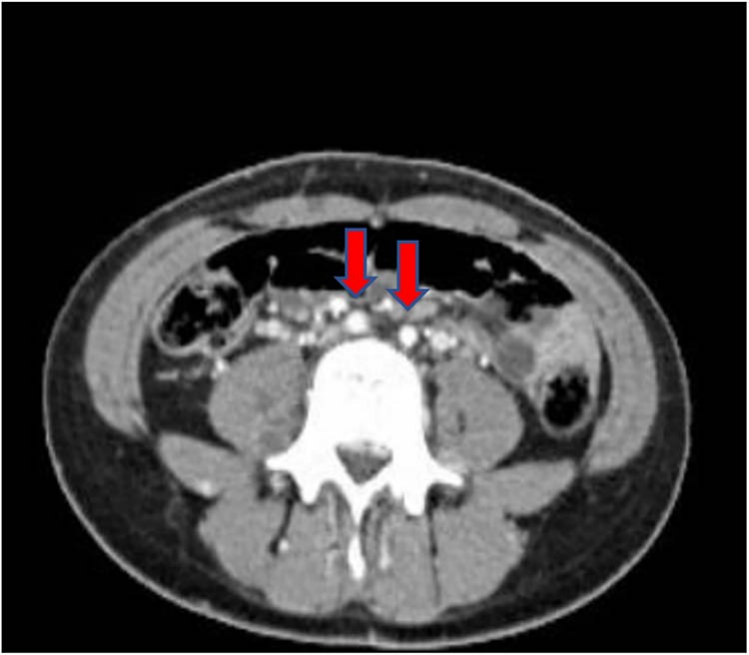
Coronal section showing both iliac arteries (red arrows) with absent iliac veins.

**Figure 3:**
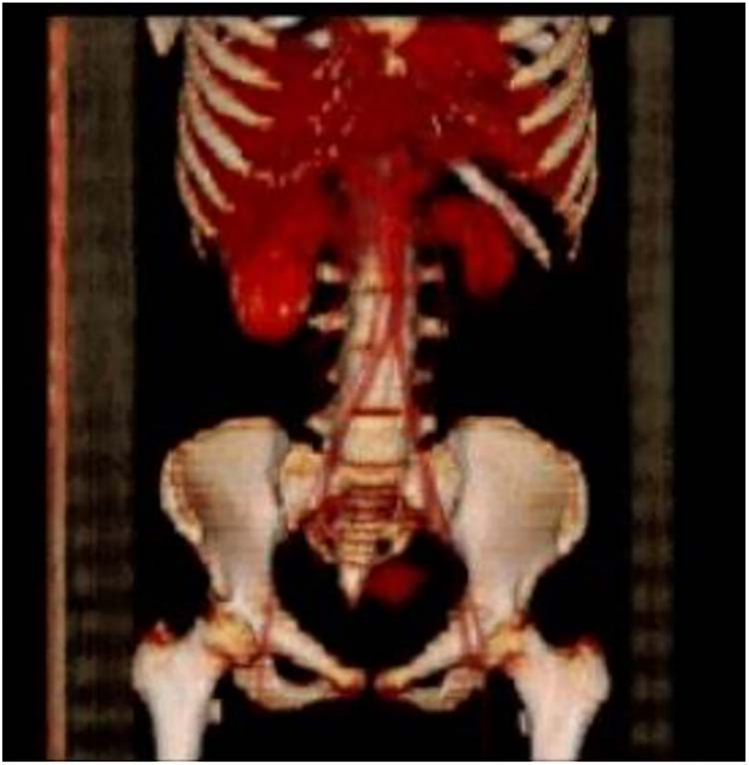
Atrophied left kidney.

**Figure 4:**
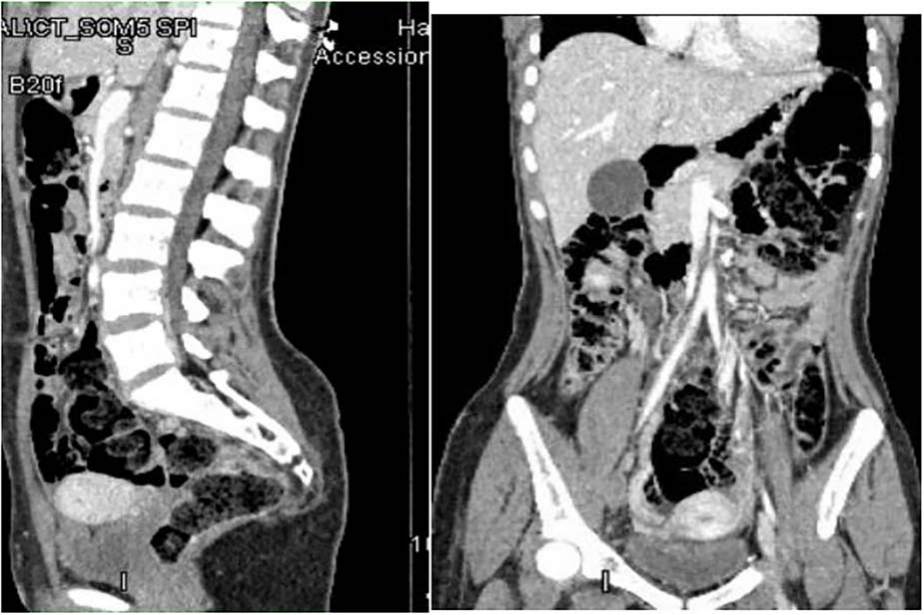
Patent abdominal aorta and iliac branches.

Computed tomography (CT) abdomen and pelvis done as part of a workup to look for an intra-abdominal extension of the thrombus showed non-visualization of IVC (Fig. 1) at the suprarenal and infrarenal levels, together with the absence of both iliac veins (Fig. 2) and subsequent dilatation of both ascending lumbar veins, which showed complete thrombosis on the right side and partial thrombosis on the left side. The lumbar veins contributed to the drainage of the lower limb veins to the azygous vein. The hepatic and suprahepatic segments of IVC were visualized, which normally drained to the heart. Atrophy of the left kidney was noted (Fig. 3). Abdominal aorta, coeliac and iliac branches were well opacified with the contrast and appeared unaffected (Fig. 4).

The case was discussed with interventional radiology for thrombectomy, and the patient underwent right iliofemoral veins, AngioJet rheolytic thrombectomy, and angioplasty.

The patient was discharged home after 4 days in stable condition, taking into account that the DVT was unprovoked and the patient had an underlying venous abnormality, being the strongest risk factor for this young, otherwise healthy lady; she has been started on warfarin, which she may need to take for the rest of her life.

## DISCUSSION

Congenital anomalies of IVC are considered a rare condition with a prevalence of 0.6% in patients with congenital heart disease and even less prevalent in healthy individuals with a percentage of 0.3%. Young patients who have IVC anomalies are at a higher risk of developing deep vein thrombosis because, despite having significant collateral veins, they have insufficient venous return and higher venous pressure in their lower extremities [1].

Absent IVC is usually incidentally found when performing cardiac procedures or investigating DVT, pulmonary embolism or arrhythmias [2]. The causes of absent IVC are thought to be developmental with failure of fusion of the main forming vessels. IVC is formed during the first weeks of embryonic life from three paired parallel veins: posterior cardinal, subcardinal and supracardinal. The heart starts to develop during this time, being the reason why some of the IVC anomalies get associated with congenital heart diseases [2–5]. Another hypothesis is that IVC thrombosis during the intrauterine or perinatal period may predispose to the agenesis of the vein [4, 6]. Venous return to the heart will be through the azygos–hemiazygos system receiving blood from the lower part of the body through the ascending lumbar veins, leading to chronic lower-extremity venous insufficiency and predisposing to DVT [7]. Patients with congenital IVC abnormalities frequently experience DVT at a young age, as in the case of our patient, compared to the average healthy population, DVT in young patients is extremely rare, with incidence 10 times lower at the age of 20–40 years [8, 9].

Generally, DVT is caused secondary to hypercoagulable conditions or venous insufficiency. Absent IVC causes venous insufficiency, which results in blood stasis within the veins, which causes spontaneous unprovoked DVT and clinical findings of venous stasis. Patients are typically present to ED with symptoms of recurrent non-healing ulcers, leg pain, and swelling, which in rare instances may cause nerve compression, paresthesia, and paralysis diagnosis is made by CT or magnetic resonance imaging [2, 8, 10]. The diagnosis was verified by a CT abdomen with contrast on our patient.

Management of DVT in a patient with absent IVC would be oral anticoagulant for life because of the increased risk of recurrence if medications are discontinued [11]. Also, catheter-directing thrombolysis is another treatment modality with multiple benefits, including minimizing bleeding risk by reducing the required systemic dose of oral anticoagulant and increasing iliofemoral patency from 36–64%, compared to anticoagulant medications alone, it also lowers the absolute risk of post-thrombolysis syndrome by 14% [12].

## CONCLUSION

In conclusion, for young patients coming with signs and symptoms of DVT, anomalies of IVC should be considered; this group is substantially more likely to have an absent IVC than the general population. Therefore, a contrast-enhanced CT scan should also be considered as part of the first workup in those patients.

## FUNDING

The publication of this article was funded by the Qatar National Library.

## CONFLICT OF INTEREST STATEMENT

No competing interests declared by the author(s).

## ETHICAL APPROVAL

The case report meets all ethical guidelines and the local legal requirements.

## DATA AVAILABILITY

The data underlying this article are available in the article and in its online supplementary material.

## CONSENT

Informed consent was taken from the patient’s guardian for publication of this case report and the images.

## GUARANTOR

Gufran Algaly.
